# Integrated multi-dimensional comparison of proteomic profiles across 54 cancer cell lines

**DOI:** 10.1016/j.isci.2025.113514

**Published:** 2025-09-05

**Authors:** Wenhao Shi, Tianlong He, Nan Wang, Annan Qian, Yuqiao Liu, Shaojun Tang, Yiying Zhu

**Affiliations:** 1 Chemistry Department, Tsinghua University, 30 Shuangqing Road, Haidian District, Beijing 100084 China; 2 Bioscience and Biomedical Engineering Thrust, Systems Hub, The Hong Kong University of Science and Technology (Guangzhou), Guangzhou 511453 China; 3 Research Center of Plastic Surgery Hospital, CAMS Key Laboratory of Tissue and Organ Regeneration, Chinese Academy of Medical Sciences and Peking Union Medical College, Beijing 100144 China; 4 Cosmos Wisdom Biotech Co. Ltd, Building 10^th^, No. 617 Jiner Road, Hangzhou 311215 China

**Keywords:** Integrative aspects of cell biology, Cancer, Proteomics

## Abstract

Human cancer cell lines are essential model systems in biomedical research. We conducted multi-level proteomics analyses on 54 widely used cancer cell lines derived from various tissues using two prominent proteomics technologies: mass spectrometry (MS) and reverse-phase protein array (RPPA). Our analysis identified 10,088 proteins, 33,161 phosphorylation sites across 7,469 phosphoproteins, and 56,320 site-specific glycans on 14,228 glycosylation sites from 5,966 glycoproteins, along with 305 drug-relevant protein and phosphoprotein targets. Analysis of this rich dataset yielded numerous biological insights, including protein features that distinguish tissue origins and cell line-specific kinase activation patterns, reflecting signaling diversity across cancer types. These findings may inform therapeutic strategies and support rational model system selection. Additionally, MS and RPPA showed consistent fold-change estimation and provided complementary views of proteome and signaling variation. This comprehensive resource facilitates biomarker discovery, signaling analysis, and translational oncology research across diverse human tumor types.

## Highlights

• Comprehensive profiling of cancer cell lines reveals total, phospho, and glycoproteomes
• Phosphoproteomics highlights cell line-specific kinase activity and potential vulnerabilities
• MS and RPPA jointly capture proteome and signaling variation with strong concordance

## Graphical abstract

**Figure:**
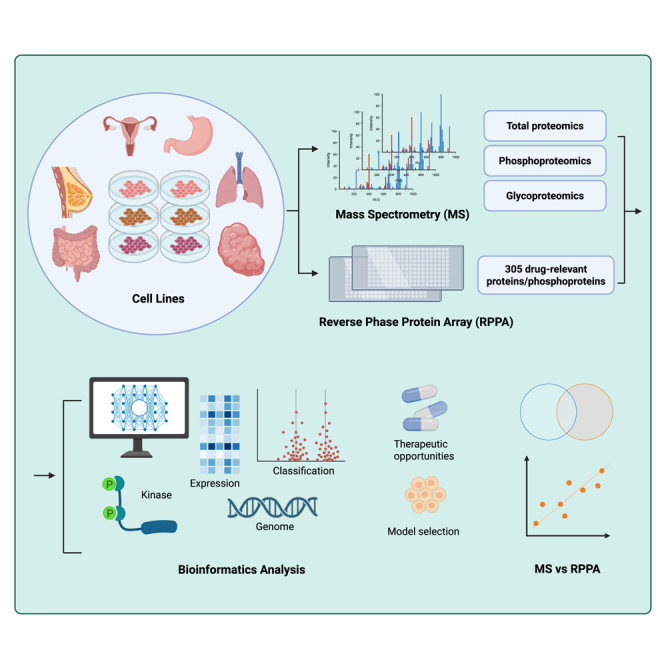

## Introduction

Human cancer cell lines serve as essential models for uncovering the molecular mechanisms and cellular behaviors associated with oncogenesis and disease progression. Past studies have leveraged various proteomics approaches to establish proteoform-oriented expression profiles in these cell lines, providing critical insights into cancer systems biology. Most of these studies utilized large-scale analyses covering proteome-wide and post-translational modification (PTM) levels, leading to the creation of databases such as the Cancer Cell Line Encyclopedia (CCLE) and the National Cancer Institute’s 60 (NCI-60) cancer cell line collection. Using 10-plex TMT quantitative proteomics, Gygi’s team profiled 375 cell lines across 22 lineages, quantifying over 12,000 proteins across wide dynamic ranges.^1^ In line with that, Aebersold’s team conducted quantitative mass spectrometry (MS) proteomics experiments on NCI-60 cancer cells using an SWATH/DIA-based approach, generating over 8,000 unique proteins, of which about 3,171 proteins were compared across cell lines.^2^ This proteotypic landscape underscores the value of protein-level data in interpreting cellular phenotypes, inferring protein coregulatory networks, and predicting proteo-transcriptomic-informed drug responses. Complementary studies on NCI-60 cell lines employed label-free MS to analyze proteome and kinome profiles, while others have used MS to characterize the phosphoproteome, illuminating the mechanisms of action (MOA) of cancer drugs.^3^^,^^4^ The DepMap Project further integrates proteomic data with pharmacological profiles, deepening our understanding of molecular vulnerabilities and therapeutic targets in cancer (https://depmap.org/portal/).

In addition to MS-based proteomics, affinity-based protein quantification methods, such as the Reverse Phase Protein Array (RPPA), allow high-throughput quantification of proteins and modified proteoforms using specific antibodies.^5^^,^^6^ Cell-based RPPA experiments, including those by our team, are commonly used to investigate biological mechanisms under perturbation.^7^^,^^8^ This technique has characterized NCI-60 cell lines, generating drug response networks and signaling pathway activity profiles that impact cellular fates.^9^^,^^10^^,^^11^ A key advantage of RPPA is its ability to quantify low-abundance proteins and PTM. For example, Davies and colleagues used a panel of 222 protein features, including total and phosphorylated proteins, to categorize NCI-60 cells into five clusters, each associated with specific mutations linked to drug response.^10^ Other studies identified protein-inferred drug response, patterning the activation/phosphorylation states of 135 proteins and defining six core cancer signaling modules related to therapeutic responses.^12^ Hundreds of cancer cell lines with more than 200 protein targets have been profiled by RPPA to date, with data repositories such as CCLE, PRIDE (https://www.ebi.ac.uk/pride/), and MD Anderson’s Cell Lines Project (MCLP: https://tcpaportal.org/mclp/#/) offering comprehensive datasets that allow researchers to investigate protein functions, drug targets, and biomarkers across cancer types.^13^^,^^14^^,^^15^ Recent RPPA studies expanded these resources, adding 447 clinically relevant dual-actionable targets in the cancer field.^16^

MS-based proteomics and RPPA each offer distinct advantages in proteomics research. MS-based proteomics digests proteins into peptides, identifies them by comparing MS/MS spectra with theoretical protein sequences, and enables the large-scale characterization of the entire proteome and modified forms, whereas affinity detection-based methods, such as RPPA, employ antibodies to measure protein levels across hundreds of samples simultaneously.^5^^,^^8^^,^^17^^,^^18^^,^^19^^,^^20^ While several studies have integrated MS and RPPA data, most focus on clinical samples. For example, MS and RPPA have been combined to provide quantitative proteomic landscapes of breast cancer tissue.^21^ Proteomics data derived from The Cancer Genome Atlas (TCGA) using RPPA (quantifying 150–200 protein forms) have also been compared with global MS proteomics from the Clinical Proteomics Tumor Analysis Consortium (CPTAC, https://proteomics.cancer.gov/programs/cptac), highlighting the role of protein-driven therapy development across multiple cancer types.^22^ Despite these advances, deep characterization of cancer cell lines using multiple proteomics methods remains limited, with only a few studies performing such analyses on a systemic level.^8^

Beyond total protein profiling, PTMs, mainly phosphorylation and glycosylation, are critical cellular function governors in cancer.^23^^,^^24^^,^^25^^,^^26^ Phosphoproteomics, which characterizes site-specific phosphorylation on proteins, is invaluable for uncovering dysfunctional kinase activities and thus aiding the development of therapeutic drugs such as kinase inhibitors.^4^^,^^17^^,^^27^^,^^28^ Databases such as PhosphoSitePlus provide extensive data on phosphorylation and other PTM data for cancer cell lines, supporting targeted research (https://www.phosphosite.org/). Similarly, glycoproteomics, which examines glycosylation modifications, has been extensively studied due to its potential as a diagnostic biomarker for cancer and other diseases.^29^^,^^30^ Recent advances in MS technology have enabled large-scale glycosylation profiling of cancer cell lines,^31^^,^^32^ offering mechanistic insights into glycosylation on a global scale during oncogenesis and disease progression.

To fill the gap in multi-dimensional proteomic analysis of cancer cell lines, we applied label-free MS together with RPPA to obtain quantitative proteomics data on 54 representative cancer cell lines, predominantly from NCI-60. This study provides a comprehensive proteomics landscape, incorporating the total proteome, phosphoproteome, and glycoproteome, which reinforces our understanding of protein-level regulation in cancer cells. By comparing MS and RPPA datasets, our study underscores the complementary strengths of these two methods: MS offers a broad view of protein abundance and PTMs, while RPPA facilitates the targeted quantification of specific proteins and PTMs, even at low abundances. This combined approach highlights the value of multi-dimensional proteomic data in discovering therapeutic targets, identifying biomarkers for cancer subtyping, and predicting cellular responses, supporting advances in precision oncology.

## Results

### Overview of the study

**Figure 1:**
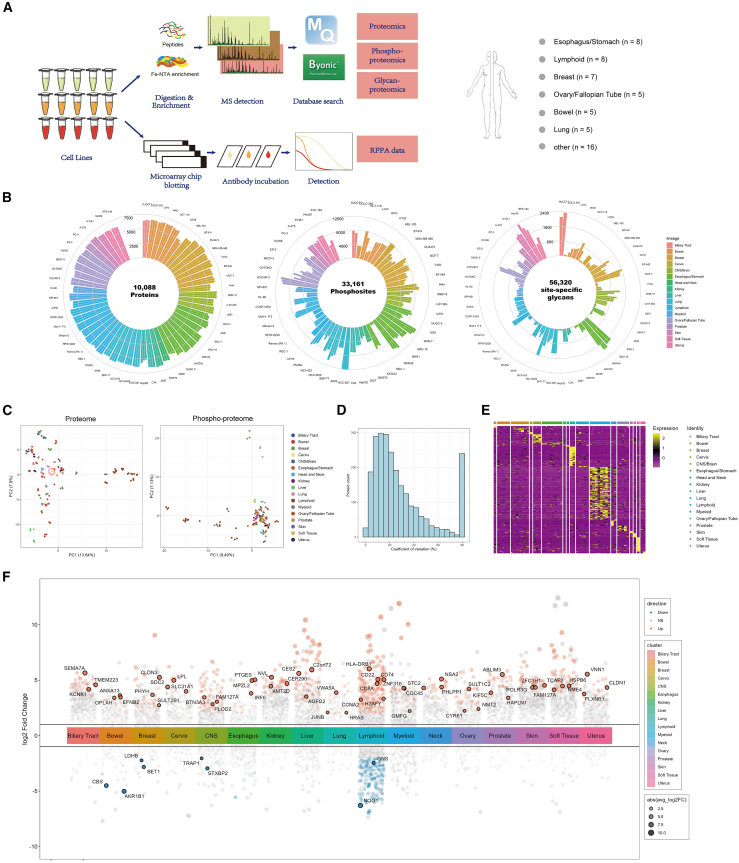
Experimental Design and Overview of the Data (A) Workflow of proteomics analysis of cancer cell lines: Cultivated cells were divided into separate aliquots for MS and RPPA sample preparation. After mass spectrometry or Array-Pro analyzer analysis, MS data was processed separately for the total proteome, phosphoproteome, and glycoproteome, while RPPA data was processed to measure protein concentrations. Cell line classification was displayed on the right based on their tissue of origin. (B) Number of detections for total proteins, phosphosites, and site-specific glycans detected across cell lines: The outer circle displays names of cell lines, accompanied by bars representing the number of detections for each cell line. The colors of the bars indicate the tissue origin of these cell lines. (C) Principal component analysis of proteome (left) and phosphoproteome (right) for cell lines: Each dot represents a sample, and the percentage value indicates the variance explained. The red circle shows three replicates from HeLa cells. (D) Distribution of protein coefficient of variation (CV) values in three technical replicates of HeLa cells analyzed by MS. (E) Heatmap of protein markers across different lineage origins: The color scheme represents standardized scores. Yellow indicates over-expressed proteins. (F) Multi-group volcano plot of differentially expressed proteins across lineages: Colored dots represent significant expression, with a Wilcoxon adjusted *p*-value of less than 0.05.

In this study, we investigated 54 widely used tumor cell lines derived from various human tissues. Cultured cells were harvested and prepared separately for MS and RPPA proteomics analysis. The MS-based analysis was more comprehensive, encompassing the whole proteome, phosphoproteome, and glycoproteome (Figure 1A). These cell lines were derived from tissues on breast (*n* = 7), esophagus/stomach (*n* = 8), lymphoid (*n* = 8), lung (*n* = 5), ovary/fallopian tube (*n* = 5), large bowel (*n* = 5), and other origins (*n* = 16) (Figure 1A; Table S1).

Using the iBAQ algorithm in MaxQuant for quantification, we detected 10,088 proteins in the MS-based dataset in total, with a median of 6,330 proteins detected per sample, while LFQ was then applied for downstream analysis, due to its suitability for comparing protein abundances across different samples. After applying the filtering criteria described in the STAR Methods, we identified 6,810 proteins in the MS-based dataset, with a median of 4,334 proteins detected per sample (Figure 1B; Table S2). In the phosphoproteomics dataset, we detected 7,469 phosphoproteins with 33,161 phosphorylation sites (location probability >0.75), and median values of ∼3,000 phosphoproteins and 6,000 phospho-sites per sample (Figure 1B; Table S3). Furthermore, we identified 56,320 site-specific glycans from 14,228 glycosylation sites, spanning 5,966 glycoproteins (Figure 1B; Table S4). The RPPA panel consisted of 231 whole-protein and 74 phosphosite-specific antibodies (Table S5). Therefore, the RPPA data constructed 305 drug-relevant protein and phosphoprotein profile targets across all cell lines.

Principal component analysis (PCA) of protein expression and phosphorylation (Figure 1C) demonstrated strong reproducibility, with a lineage-dependent pattern in the cell line replicates, generating consistent and biologically meaningful data. Although phosphorylation reflects the cell’s different states and can vary under external perturbations; in this study, our data represent cells under their steady state condition and thereby their basal phosphorylation levels. Similarly, the phosphoproteomics again revealed significant differences based on their tissue origin, with a clear distinction between cell lines derived from nonsolid and solid tumors, strengthening the rigor of the data (Figure 1C). As for the glycan modification, since they varied significantly among different cell lines (Figure 1B), glycosylation data were not shown in dimension reduction analysis. We then randomly picked the HeLa cell line to calculate the coefficient of variation (CV), that were quantified based on three replicate samples across proteins. We observed that over 80% of proteins exhibited a CV below 20%, indicating high technical reproducibility of the data measured through MS (Figure 1D).

We constructed a heatmap based on the total protein expression by highlighting the most diversely expressed proteins across cell lines, without hierarchical clustering (Figure 1E). We also conducted tissue-level differential expression analysis based on featured marker proteins (COSG scores >0.5), resulting in a total of 292 targets being identified (Figure 1F; Table S2). As expected, the identified markers reflected distinct protein expression patterns across multiple tissue-derived cell lines, aligning with known cancer biology. For example, upregulated proteins such as HLA-DRB1, CD22, CD74, and CD8A are consistent with established roles in lymphoid malignancies and oncogenic signaling, supporting prior studies identifying CD74 as a diagnostic and therapeutic target in lymphomas.^33^^,^^34^^,^^35^ Downregulated proteins, including NQO1 and LDHB, align with literature describing metabolic shifts in cancer, such as NQO1 loss contributing to oxidative stress resistance^36^ and LDHB suppression altering glycolytic pathways in breast cancers.^37^ The presence of histone methyltransferases such as KMT2D reinforces previous findings linking epigenetic dysregulation and cell cycle control to cancer progression.^38^ Taken together, the findings corroborated with existing cancer proteomics data, providing additional insights that may guide further biological discovery. Thus far, our comprehensive proteomics data provides multi-layered resources from total proteome, phosphoproteome, and glycoproteome perspectives across a diverse set of cancer cell lines, offering valuable insights into cancer biology and the molecular underpinnings of cell line-specific behaviors.

### Phosphoproteomics revealed phosphorylation dynamics of cancer cell lines

**Figure 2:**
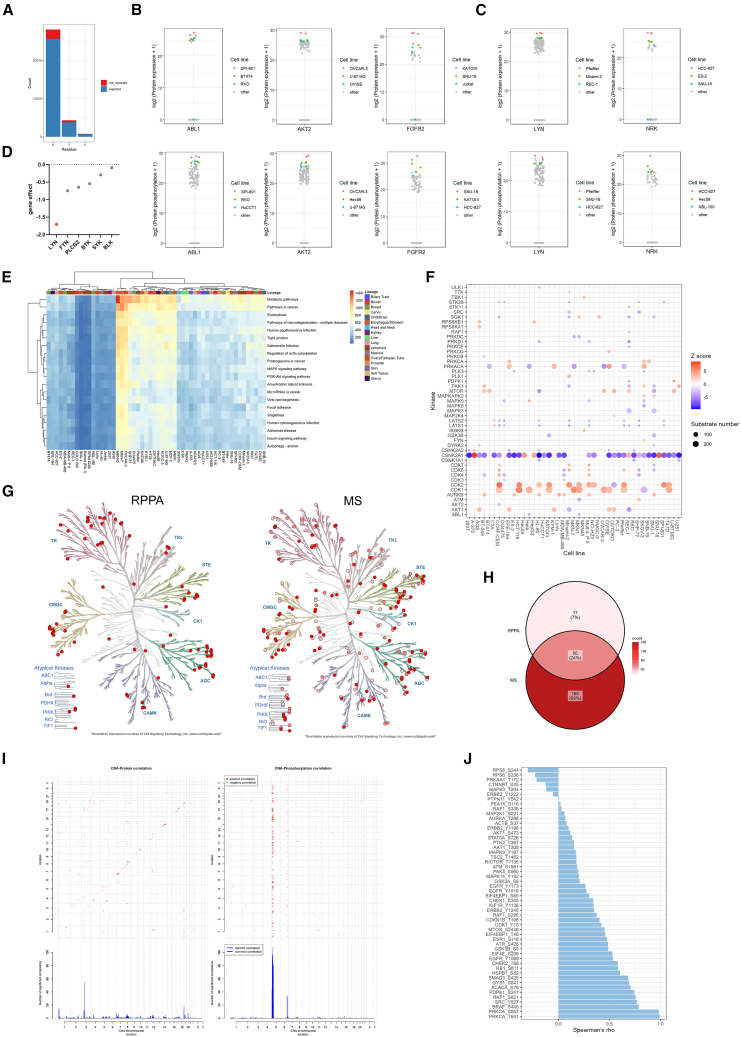
Phosphoproteome Landscape of Cell Lines (A) Distribution of phosphorylation types: This panel illustrates the distribution of phosphorylation events across serine, threonine, and tyrosine residues. The modifications (uncurated) are compared against the PhosphoSite Plus database (www.phosphosite.org), with reported modifications shown in blue and unreported modifications in red. (MS data). (B) Whole proteome and site-specific phosphorylation of ABL1 (P00519) and AKT2 (P31751) in cell lines: The abundance of both ABL1 and AKT2 proteins and their phosphorylation levels in the SPI-801 and OVCAR-3 cell lines are displayed. Protein abundance is calculated as the sum of the top three unique peptides, while phosphorylation abundance is determined by the sum of all identified phosphopeptides. (MS data). (C) Protein expression and site-specific phosphorylation levels of LYN and NRK across cell lines. The top three most abundant cell lines are shown. (MS data). (D) Gene dependency scores of kinases involved in the BCR signaling pathway. (E) Heatmap of signaling pathways in cell lines: The phosphorylation levels of proteins across various cell lines are shown, with the number of detected phosphorylation sites representing these levels. The block colors indicate pathway enrichment, and the top of the heatmap is color-coded to represent the tissue origins of the cell lines. (MS data). (F) Kinase activity inferred from substrates: Kinase activity is depicted based on the number of phosphorylated substrates across different cell lines. The circle size corresponds to the number of phosphorylated substrates for each kinase, and the color intensity reflects the Z scores, representing the relative abundance of phosphorylated substrates. (MS data). (G) Kinase detection mapped onto the human kinome tree for RPPA (left) and MS (right); node color intensity reflects detection frequency (tree adapted from KinMap based on Cell Signaling Technology classification). (H) Venn diagram showing the overlap of kinase identifications between MS and RPPA platforms. (I) Correlation between copy number alterations (CNA) and protein or phospho-protein levels across cell lines. (MS data). (J) Correlation between site-specific phosphorylation and total protein abundance across cell lines. (RPPA data).

Phosphorylation, a key PTM, plays a crucial role in regulating cellular signaling pathways. Oncogenesis is often linked to dysregulated molecular signaling, and therefore, understanding phosphorylation patterns can provide valuable insights into cancer mechanisms and therapeutic targets. As of January 2024, the FDA has authorized 80 small-molecule kinase inhibitors targeting 24 kinases that regulate protein phosphorylation on serine, threonine, and tyrosine residues, with more therapeutics under development.^23^^,^^24^ Studying phosphorylation patterns of human cancer cell lines may help estimate the disease mechanisms, select suitable models, and evaluate drug responses.^4^ In our MS phospho-proteome analysis, we identified 33,161 phosphorylation sites, with 90.9% of them previously reported in the PhosphoSite Plus database (www.phosphosite.org) (Figure 2A). Due to the use of immobilized metal affinity chromatography (IMAC) for phosphopeptide enrichment, the distribution of serine, threonine, and tyrosine phosphorylation in this study mirrored the natural occurrence of these PTMs, with serine and threonine being common and tyrosine being the least prevalent (Figure 2A).

To systematically identify candidate kinase drivers, we screened for kinases with both total protein and phosphorylation levels at least 1.5-fold higher in one cell line than in any other, and required that the lowest replicate still exceeded 50% of the highest replicate in the rest. This stringent criterion enriches for kinases that are not only hyperphosphorylated but also highly expressed—indicating a higher likelihood of functional activation and oncogenic relevance. This approach successfully recovered known kinase drivers (Figure 2B). For example, ABL1 was selectively activated in the Chronic myeloid leukemia (CML) cell line SPI-801, which was consistent with BCR-ABL1–driven signaling and imatinib sensitivity.^27^ Similarly, AKT2 was strongly upregulated in OVCAR-3, in line with its role in ovarian cancer cell survival and drug resistance.^39^^,^^40^ We also identified FGFR2 as a context-dependent candidate, with marked activation in KATOIII and SNU-16 (gastric carcinoma). These findings align with known FGFR2 amplification in gastric cancer^41^^,^^42^ and therapeutic responses to FGFR2b inhibitors such as bemarituzumab.^43^ This method provides an effective framework to prioritize actionable kinases based on integrated proteomic activation signals.

Given that known oncogenic kinases were successfully identified through this level ranking analysis across various cell lines, we believe that other top 1-ranking kinases we identified may represent previously underexplored targets (Figure 2C). Notably, LYN was specifically upregulated in the Pfeiffer cell line, a diffuse large B-cell lymphoma (DLBCL) model driven by aberrant B cell receptor (BCR) signaling (Figure 2C). Although prior studies have primarily focused on BTK inhibition in this context,^44^ our analysis revealed that LYN exhibited the lowest RNAi gene effect score among BCR pathway kinases (Figure 2D), based on CCLE dependency data, indicating that LYN inhibition may have a stronger effect on cell viability than BTK in this model. These findings highlight how phosphoproteomic profiling can inform both the prioritization of known therapeutic targets and the discovery of context-specific vulnerabilities. We further investigated NRK (Nik-related kinase) as a potential therapeutic target. NRK protein and phosphorylation levels were markedly elevated in a subset of cell lines, particularly HCC-827 (lung adenocarcinoma) (Figure 2C). External databases, including The Human Protein Atlas and PhosphoSitePlus, confirmed NRK overexpression and phosphorylation in this line. Although NRK has been detected in global phosphotyrosine profiling studies,^28^^,^^45^ it remains poorly characterized. These studies linked NRK to signaling downstream of oncogenic EGFR and c-Met, suggesting it may define a subtype of lung cancer with distinct kinase dependencies.

The other top cell lines, aside from the first-ranked one, may also provide valuable insights. For example, in Figure 2B, ABL1 is highly phosphorylated in RKO and HuCCT1, suggesting potential roles in colorectal and cholangiocarcinoma contexts where ABL1 is less well characterized. Similarly, AKT2 is elevated in U-87 MG and OVISE, indicating possible isoform-specific dependencies in glioblastoma and ovarian clear cell carcinoma. FGFR2 phosphorylation and expression in HCC-827 and Jurkat may reflect signaling plasticity or unexplored functional roles in lung and hematologic cancers. These findings highlight underexplored kinase dependencies worthy of further investigation.

We also generated a heatmap of signaling pathways across different cell lines (Figure 2E), showing no clear correlation between tissue origin and phosphorylation patterns. This was consistent with previous findings that tumors, even originating from the same tissue, require personalized treatments.^46^^,^^47^ Notably, hyperphosphorylation of the PI3K/Akt/mTOR pathway was observed in the gastric cancer cell lines MKN45 and MKN7, consistent with reports that these cell lines are sensitive to inhibitors targeting this pathway from the CancerRxGene database (www.cancerrxgene.org/celllines).

Further, we performed a kinase-substrate enrichment analysis (KSEA), represented through a dot plot (Figure 2F). Noteworthy findings included the high activity of CDK6 substrates in the Pfeiffer cell line, a diffuse large B-cell lymphoma line, aligning with reports that CDK4/6 inhibitors are effective against aggressive B-cell lymphomas.^48^ In addition to tumor types that have been previously reported to have CDK4/6 as a potential therapeutic target, some other tumor types also displayed signs of CDK4/6 activation. For example, in the CCRF-CEM cell line derived from T cell acute lymphoblastic leukemia, substrates of CDK4 and CDK6 showed significant phosphorylation (Figure 2F), as previously described.^49^^,^^50^ We also noted the overexpression of CSNK2A1, a subtype of CK2, across multiple cancer cell lines, including SNU-16 (gastric adenocarcinoma) and HepG2 (hepatoblastoma). CK2 is known to be overexpressed in various cancers, and inhibitors such as CX-4945 show therapeutic potential in gastric and liver cancers.^51^^,^^52^^,^^53^

We compared kinase detection between MS and RPPA using kinase tree visualizations and detection counts (Figures 2G and 2H). MS provided broader kinome coverage, detecting a total of 216 kinases across nearly all families, albeit with lower detection depth per kinase. In contrast, RPPA identified 73 kinases with higher sensitivity at select phosphorylation sites, particularly within the tyrosine kinase (TK) family, driven by a panel of 74 phospho-site–specific antibodies. Of the kinases detected by RPPA, 77% overlapped with those detected by MS (56 shared kinases), highlighting RPPA’s focused but biologically relevant coverage. These findings align with the kinase tree results, where MS captured a more diverse kinome landscape, while RPPA concentrated on key regulatory nodes. Together, these data confirm the complementary nature of the two platforms: MS enables comprehensive kinome profiling, while RPPA delivers sensitive, targeted quantification of clinically significant phosphosites.

We then assessed the effects of copy number alterations (CNA) on both the proteome and phosphoproteome (Figure 2I). As expected, *cis*-regulation—where CNAs influence the expression of genes located at the same chromosomal locus—was more pronounced at the proteomic level than at the phosphoproteomic level. CNA effects on protein abundance were broadly distributed across the genome, while CNA-phosphorylation correlations were more spatially restricted, with concentrated signals observed on chromosomes 5p and 6q. Since phosphorylation levels were normalized to the corresponding total protein abundance, these associations likely reflect CNA-driven alterations in kinase or phosphatase activity rather than changes in protein levels alone. However, it is also possible that phosphoproteomic data introduce technical biases that could contribute to these patterns. Factors such as enrichment efficiency, peptide ionization properties, or uneven coverage of phosphosites across the genome may influence detection sensitivity. Additionally, frequent CNA events or overrepresentation of highly phosphorylated proteins in certain chromosomal regions could artificially amplify apparent CNA-phosphorylation associations. In contrast, CNA-driven protein-level regulation appeared more widespread, including known *trans*-acting effects on proteins encoded on chromosomes 3p and 19p. While the observed phosphoproteomic hotspots may suggest signaling hubs encoded on 5p and 6q, further validation will be necessary to distinguish true biological regulation from potential measurement artifacts.

Phosphorylation levels are not directly correlated with total protein levels under activated conditions for cellular signaling, which is influenced by the modulation of kinases and phosphatases. At the basal level for the tested cell lines, we observed that the majority of protein phosphorylation at critical sites showed strong correlations (r > 0.6), moderate correlations (0.4 < r < 0.6), and weak correlations (0.2 < r < 0.4) with protein levels (Figure 2J). This suggests that the overexpression of certain proteins may correspond to relatively higher phosphorylation levels, which is significant for analyzing the activation of signaling pathways based on total proteomics data. However, exceptions exist where no correlation, or even a reverse correlation, exists between protein expression and phosphorylation levels (Figure 2G, r < 0.2). This indicates that, at the basal level, the associated signaling pathways can be strongly activated through mechanisms other than the overexpression of proteins.^54^^,^^55^

Our phosphoproteomics analysis provides a comprehensive view of kinase activities, activated signaling pathways, and the relationships between total protein levels and phosphorylation. These insights are valuable for selecting appropriate cancer cell line models for drug and cellular signaling research and for predicting the sensitivities of cancer cell lines to kinase-targeting therapies. This understanding can help identify key kinase-driven processes and improve the precision of therapeutic interventions in cancer research.

### Glycoproteome landscape of cancer cell lines

Protein glycosylation is a complex PTM that significantly influences protein structure, stability, function, and intracellular signaling.^56^ Recent advancements in mass spectrometry and computational tools have enabled the large-scale analysis of intact glycopeptides. The IMAC can enrich glycopeptides carrying sialic acids, as well as phosphorylated glycans such as mannose-6-phosphate and extra acidic amino acid peptides.^57^

**Figure 3:**
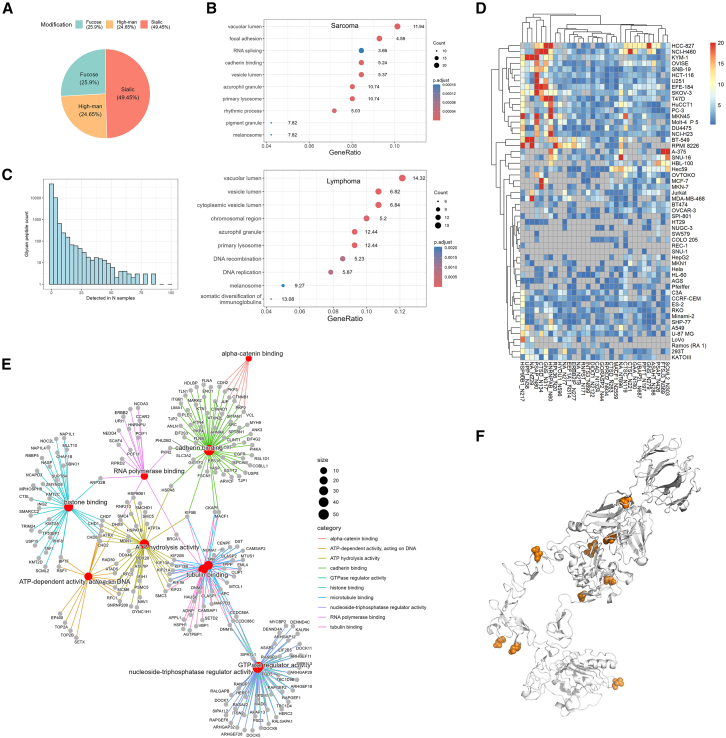
Glycoproteome Landscape of Cell Lines (A) Distribution of glycosylation types: Site-specific glycans were classified into sialic acid, fucose, and high-mannose types. A pie chart displays the percentage distribution of these glycan types across the cell lines. (B) Functional enrichment of glycoproteins in sarcoma (top) and lymphoma (bottom), based on GO biological process analysis. (C) Distribution of site-specific glycans: The Y axis shows the counts of site-specific glycans identified by MS in the cell lines, while the X axis represents the number of samples in which each glycopeptide was detected. (D) Heatmap of glycoprotein abundance across diverse cancer cell lines. (E) Network representation of glycoproteins involved in enriched molecular functions. (F) Summary of detected glycosylation sites on EGFR, aggregated across all analyzed cell lines.

This study characterized 56,320 site-specific glycans at 14,228 glycosylation sites from 5,966 glycoproteins. Among the identified glycans, 49.72% contained sialic acid, 25.11% were high-mannose types, and 25.16% contained fucose (Figure 3A). To explore functional implications, we performed Gene Ontology (GO) enrichment analysis of glycoproteins frequently identified in more than 30% of sarcoma and lymphoma cell lines (Figure 3B). Despite their differing tissue origins, both cancer types shared vacuolar lumen as the most significantly enriched term, indicating common trafficking and endocytic regulation pathways.

Glycosylation in various tumor cell lines exhibits significant heterogeneity (Figure 3C). Only 5,203/14,228 (36.6%) of glycosylation sites are consistently detected (defined as identified in more than half of the replicates) in at least one cell line, and only 21/14,228 (0.15%) are detected across 27 cell lines. We therefore investigated glycoprotein expression across 27 tumor cell lines using unsupervised hierarchical clustering (Figure 3D). Distinct lineage-specific patterns emerged: epithelial-derived lines (e.g., HCC-827, NCI-H460, OVISE) showed higher overall glycoprotein abundance, particularly in cadherin-binding and ECM-associated proteins, whereas hematologic lines (e.g., CCRF-CEM, Pfeiffer, Ramos) displayed lower glycoprotein detection, reflecting differences in surface glycan presentation and enrichment efficiency.

Key glycoproteins implicated in these pathways included EGFR, CLINT1, ESYT2, ITGB1, EIF4G2, PLP4, TNND1, and PICALM—all known to undergo extensive glycosylation (Figure 3E). Notably, Epidermal Growth Factor Receptor (EGFR), a receptor tyrosine kinase and an important target for cancer therapies, was found to have multiple glycosylation sites (N1044, N1094, N128, N352, N361, N413, N444, N528, N592, N603, N623) (Figure 3F). Among these, N361 and N352, located within the extracellular domain of EGFR, have been previously reported to be essential for maintaining EGF binding sites.^30^^,^^58^^,^^59^ Additionally, N528 exhibits significant glycosylation, with 73 site-specific glycans and 44 different glycan types identified at this site. These modifications may play a critical role in the structure and function of EGFR, influencing its activity and interactions.

Our glycoproteomic analysis reveals the diverse and lineage-specific glycosylation patterns across cancer cell lines, offering valuable insights into key drug targets and signaling pathways. This comprehensive profiling provides a powerful framework for understanding cancer cell behavior at the molecular level and lays the groundwork for future studies investigating the functional role.

### Comparison of mass spectrometry and reverse-phase protein array proteomics data

**Figure 4:**
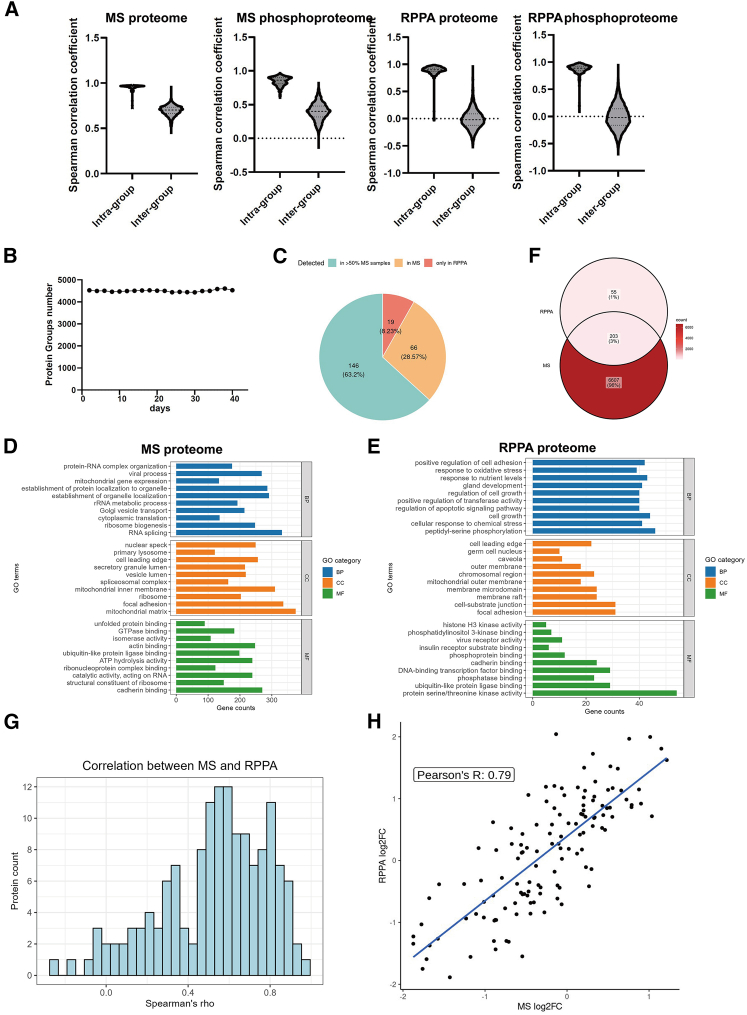
Comparison between RPPA and MS Whole Proteomics Data (A) Spearman correlation coefficients: Comparison of intra- and inter-group correlations for MS whole proteome, MS phosphoproteome, RPPA whole proteins, and RPPA phosphorylation sites, shown from left to right. (B) MS instrument monitoring: Daily tracking of MS performance using whole-cell lysate (HeLa cells) tryptic digest standards. (C) Protein detection comparison: Analysis of 231 proteins in the RPPA panel. Cyan indicates proteins detected by MS in more than 50% of samples, orange for proteins detected in fewer than 50%, and dark orange for proteins not detected by MS. (D) GO term enrichment of proteins identified by MS, categorized by biological process (BP), cellular component (CC), and molecular function (MF). (E) GO term enrichment of proteins identified by RPPA, categorized by biological process (BP), cellular component (CC), and molecular function (MF). (F) Overlap of protein identifications between MS and RPPA platforms, shown as a Venn diagram. (G) Protein quantitation correlation: Spearman’s R-values indicate the relationship of protein levels between MS and RPPA data. The X axis represents R-values, while the Y axis shows the count of proteins within each range. (H) Fold change correlation: Comparison of protein expression fold changes across different cell lines as measured by MS and RPPA, based on datasets from myeloid and tumor epithelial cells.

The comparison between MS and RPPA proteomics data reveals key insights into their performance and correlation. The Spearman correlation coefficients (Figure 4A) show high intra-group consistency for both technologies, with values ranging from 0.85 to 0.95, indicating strong reproducibility within the same experimental conditions. Inter-group correlations are notably weaker, especially in phosphorylation datasets, where MS phosphoproteome inter-group correlations are 0.40, and RPPA phosphorylation sites are -0.007, showing greater variability across different cell lines. MS performance was validated daily using a HeLa cell lysate digest as a reference standard (Figure 4B), ensuring consistent signal intensity and retention time stability throughout the study. MS provides a detailed and comprehensive analysis of proteins and PTMs, including phosphorylation. However, it can exhibit higher variability between experimental groups and have missing data points. Conversely, RPPA is more standardized and reproducible, especially for high-throughput applications.

In this study, we used RPPA technology to analyze 231 proteins. Out of these, 212 proteins (91.8%) were successfully quantified by MS, but 19 proteins (8.2%) were missed by MS (Figure 4C). Due to missing values in the MS data, only 146 proteins (∼63.2%) were quantified by MS in more than half of the samples, while 66 proteins (28.6%) were quantified in fewer than half. We first compared the functional classification of proteins detected by each platform (Figures 4D and 4E), followed by a Venn diagram analysis to evaluate the overlap between MS and RPPA protein identifications (Figure 4F). Notably, the molecular functions of the detected proteins differed between the two methods: proteins identified by MS were enriched for cadherin binding, whereas those detected by RPPA were predominantly associated with protein serine/threonine kinase activity. Despite the differing detection mechanisms, both platforms identified a shared set of 6,865 proteins, with only 55 proteins (∼1%) uniquely detected by RPPA. This comparison underscores the distinct yet complementary nature of MS and RPPA. It is particularly notable that these two platforms—based on fundamentally different principles—can still achieve meaningful concordance in protein identification and biological insight, which highlights the robustness and integrative value of combining both technologies.

To assess quantitative consistency, we compared expression levels across platforms. Notably, 90% of the proteins showed a positive correlation between the two methods, with a median correlation coefficient of 0.6 (Figure 4G), indicating strong agreement between the technologies. To minimize method-specific bias, we focused on fold-change comparisons rather than absolute abundance values. Based on PCA results, we identified two biologically distinct groups—tumor epithelial and myeloid cell lines—and calculated fold changes between them for both methods. The resulting inter-method correlation for protein fold changes reached 0.79 (Figure 4H), exceeding the correlation based on raw values. These findings support the robustness of both MS and RPPA for quantifying biologically meaningful protein differences.

In summary, MS and RPPA provide complementary insights into proteomics data. MS offers broad proteome and PTM coverage, albeit with some missing data, while RPPA delivers more focused, consistent data based on antibody availability. Together, these platforms validate and enhance each other, supporting robust and reliable protein quantification across diverse samples.

## Discussion

Previous studies have extensively profiled the whole proteome landscape of pan-cancer cell lines, offering valuable insights for biological research. However, these studies have largely overlooked the role of PTMs such as phosphorylation and glycosylation, which are critical for processes such as signal transduction, tumor progression, and drug responses. Our study addresses this gap by presenting a comprehensive, multi-dimensional proteomic resource across 54 widely used cancer cell lines, integrating total proteome, phosphoproteome, and glycoproteome data.

To enrich phosphorylated and glycosylated peptides, we employed IMAC, which captures phosphopeptides and sialic acid–containing glycopeptides under acidic conditions.^31^^,^^32^^,^^57^ Although optimized for phosphopeptides, our method also yielded thousands of site-specific glycans across diverse proteins. While not exhaustive, this co-enrichment approach offers a practical means of generating glycoproteomics data without additional instrument time or complex workflows. We further developed a spectral counting–based method for quantifying site-specific glycans from Byonic search results, enabling relative comparisons across cell lines—a tool now made publicly available for the community (Data S1).

We also systematically compared MS and RPPA technologies. MS enables global, unbiased detection of thousands of proteins and PTMs, while RPPA offers sensitive, targeted quantification of specific phosphosites—particularly within tyrosine kinase signaling pathways. Despite their distinct mechanisms, we observed strong concordance in protein fold-change trends between MS and RPPA, reinforcing the robustness of differential expression patterns across platforms. Together, these platforms offer complementary insights, with MS providing depth and discovery power, and RPPA enabling reproducibility and clinical scalability.

Importantly, the multi-layered dataset enabled us to identify context-specific kinase activation events with potential therapeutic relevance. For instance, LYN, a Src-family kinase, was selectively activated in the Pfeiffer DLBCL cell line—highlighting a vulnerability beyond the canonical BTK axis. Similarly, NRK emerged as a poorly characterized but highly phosphorylated kinase in HCC-827 lung adenocarcinoma cells, suggesting a subtype-specific dependency. These examples demonstrate how integrative proteomic profiling can uncover underexplored, actionable targets that may not be evident from genomics alone.

Beyond individual discoveries, our dataset captures lineage-specific signaling, phospho-regulatory variation, and glycosylation heterogeneity—dimensions often missed in transcriptomic or genomic analyses. While our 54-cell-line panel cannot fully represent every tumor subtype, it spans major lineages and includes extensively used experimental models. As such, it serves as a proteomic reference for model selection, pathway interrogation, and hypothesis generation in drug discovery and functional validation studies.

In summary, this work delivers a high-quality, multi-omic resource that broadens our understanding of protein-level regulation in cancer. It highlights how integrated proteomics can reveal non-genetic vulnerabilities, stratify signaling states, and guide the identification of therapeutic opportunities beyond the limits of traditional molecular profiling.

### Limitations of the study

While our study provides a high-quality, multi-layered proteomic resource across 54 diverse cancer cell lines, several limitations remain. The use of data-dependent acquisition (DDA), though optimal for post-translational modification profiling, introduces missing values that may obscure low-abundance or transient events. Glycoproteomic coverage was limited by IMAC co-enrichment, which favors acidic and sialylated glycans and may miss other glycoforms detectable by specialized enrichment methods such as lectin or HILIC. Although the cell line panel spans major tumor types, the limited number of lines per cancer subtype restricts the representation of intratumoral heterogeneity. Furthermore, this study does not propose a new computational method or modeling framework. The absence of integrated genomic or transcriptomic data also constrains the depth of systems-level insight. These limitations highlight opportunities for future studies to expand cell line diversity, adopt complementary acquisition strategies, validate functional hypotheses, and integrate multi-omic analyses to enhance mechanistic discovery and translational relevance.

## Resource availability

### Lead contact

Further information and requests for resources and reagents should be directed to and will be fulfilled by the Lead Contact, Yiying Zhu (yiying_zhu@mail.tsinghua.edu.cn).

### Materials availability

This study did not generate new unique reagents.

### Data and code availability

The mass spectrometry proteomics data have been deposited to the ProteomeXchange Consortium via the PRIDE partner repository with the dataset identifier PXD056716 (http://proteomecentral.proteomexchange.org/cgi/GetDataset?ID=PXD056716). All processed data and source code used in this study are provided as supplemental information.

## Acknowledgments

We thank Fynn Biotechnologies Co., Ltd. (Shandong, China) for providing valuable cell lines and conducting the RPPA. We also thank PRECEDO Biotechnologies (Hefei, China) for providing some of the cell lines used in this work. The graphical abstract was created with bioRender.com.

This study is supported by the Innovation Funding from the Office of Laboratory Management at 10.13039/501100004147Tsinghua University (Y. Z.).

## Author contributions

Y.Z. conceived the study. W.S. and N.W. acquired MS and RPPA proteomics data. T.H. contributed to data analysis, integration, and interpretation (initiated the project at Affiliation 2 and completed it at Affiliation 3), and W.S., A.Q., and Y.L. also assisted in these tasks. Y.Z., N.W., and S.T. supervised the bioinformatics analysis. Y.Z., W.S., and T.H. wrote the manuscript, and Y.Z. and N.W. edited it.

## Declaration of interests

The authors declare no conflict of interest.

## STAR★Methods

### Key resources table

**Table:** 

REAGENT or RESOURCE	SOURCE	IDENTIFIER
**Biological samples**		

Human cancer cell lines (n=54)	ATCC; CCTCC; Fynn Biotechnologies; PRECEDO Biotech	See Table S1

**Chemicals**		

Urea (≥99.5%)	Sigma-Aldrich	Cat# U5128
Tris-HCl, pH 8.0	Thermo Fisher Scientific	Cat# 15568025
Dithiothreitol (DTT)	Thermo Fisher Scientific	Cat# R0861
Iodoacetamide (IAA)	Sigma-Aldrich	Cat# I1149
Trypsin Gold, MS Grade	Promega	Cat# V5280
BCA Protein Assay Kit	Thermo Fisher Scientific	Cat# 23225
Fe-NTA Phosphopeptide Enrichment Kit	Thermo Fisher Scientific	Cat# A32992
Acetonitrile, LC-MS grade	Thermo Fisher Scientific	Cat# A955-4
Formic Acid, LC-MS grade	Thermo Fisher Scientific	Cat# A117-50
Trifluoroacetic Acid (TFA)	Thermo Fisher Scientific	Cat# A116-50
Beta-Mercaptoethanol	Sigma-Aldrich	Cat# M6250
Glycerol	Sigma-Aldrich	Cat# G5516
Microcon Ultracel YM-30 Filter	Millipore	Cat# MRCF0R030

**Other**		

ONCYTE SuperNOVA Slides	Grace Bio-Labs	Cat# 305173
Antibody panel (RPPA; 305 targets)	Proprietary; targets listed in Table S5	—
Quanterix Pin Tool Printer	Quanterix	Model 2470
DAKO Link 48 Autostainer	Agilent	Model Link 48
Orbitrap Eclipse Mass Spectrometer	Thermo Fisher Scientific	Model Eclipse

**Deposited data**		

Mass spectrometry raw data	ProteomeXchange	Accession: PXD056716 http://proteomecentral.proteomexchange.org/cgi/GetDataset?ID=PXD056716
Processed data and code	This paper	supplemental information

**Software and algorithms**		

MaxQuant v1.6.17.0	Cox Lab	https://www.maxquant.org
Byonic v5.0.3	Protein Metrics	https://www.proteinmetrics.com
Seurat v5.0.1	Satija Lab	https://satijalab.org/seurat
DEP v1.16.0	Bioconductor	https://bioconductor.org/packages/DEP
COSG v0.9.0	Loosolab (GitHub)	https://github.com/loosolab/cosg
clusterProfiler v4.10.0	Bioconductor	https://bioconductor.org/packages/clusterProfiler
org.Hs.eg.db v3.18.0	Bioconductor	https://bioconductor.org/packages/org.Hs.eg.db
KSEAapp v0.99.0	GitHub (casecpb)	https://github.com/casecpb/KSEAapp
multiOmicsViz v1.26.0	Bioconductor	https://bioconductor.org/packages/multiOmicsViz
R v4.3.2	R Core Team	https://www.r-project.org

### Experimental model and study participant details

#### Human cancer cell lines

Fifty-four human cancer cell lines were used in this study. Cell lines were cultured under standard conditions in a humidified incubator at 37°C with 5% CO_2_. Culture media consisted of 90% basal medium supplemented with 10% fetal bovine serum (FBS) and 1× penicillin-streptomycin (100 U/mL penicillin and 100 μg/mL streptomycin). DMEM was used for HEC59, OC316, A-375, U251, HT29, HS578T, SNB-19, MDA-MB-231, and HepH2; RPMI-1640 for OAW42, OVCAR-3, NCI-H23, T47D, HCC1954, HeyA8, MCF-7, K-562, PEO, HL-60, IGROV-1, Jurkat, NCI-H460, EFE-184, OAW28, BT474, and AGS; McCoy's 5A for ES-2, FU-OV-1, SKOV-3, and SKBR-3; and L15 medium for MDA-MB-468. All cell lines were authenticated via short tandem repeat (STR) profiling. Full names and tissue origins are listed in Table S1.

### Method details

#### Cell harvest and protein extraction

As previously described,^8^^,^^60^ once adherent cells reached a minimum confluency of 80%, they were carefully washed twice with ice-cold phosphate-buffered saline (PBS; pH 7.2, GIBCO) to remove residual media and serum components that could interfere with downstream proteomic analysis. Cells were then harvested by gentle scraping on ice to minimize proteolytic degradation and preserve protein integrity. The cell suspensions were transferred to pre-chilled tubes and centrifuged at 500 × g for 5 minutes at 4 °C to pellet the cells. After centrifugation, the supernatant was carefully aspirated to remove excess PBS, and the cell pellets were snap-frozen in liquid nitrogen or immediately stored at –80 °C until further processing. This procedure was standardized across all cell lines to ensure consistency in sample handling prior to protein extraction and mass spectrometry–based analysis.

#### Protein extraction and trypsin digestion for MS analysis

Cells were minced and lysed in lysis buffer (8 M urea, 100 mM Tris hydrochloride, pH 8.0) containing protease and phosphatase inhibitors (Thermo Scientific) followed by 1 min of sonication (3 s on and 3 s off pulse, amplitude 25%). The lysate was centrifuged at 14,000 × g for 10 min, and the supernatant was collected as whole tissue extract. Protein concentration was determined by the bicinchoninic acid (BCA) protein assay. Protein extracts (1 mg per sample) were subjected to reduction with 10 mM dithiothreitol at 56°C for 30 minutes, followed by alkylation using 10 mM iodoacetamide at room temperature in darkness for another 30 minutes.

Then, samples were digested using the FASP (filter-aided proteome preparation) method with trypsin. In brief, samples were placed in a 30 kDa Microcon filter (Millipore) and centrifuged at 14,000 × g for 20 minutes. The retained precipitate on the filter was subsequently washed twice by adding 300 μL of washing buffer (8 M urea in 100 mM Tris, pH 8.0), followed by centrifugation at 14,000 × g for another 20 minutes. Trypsin was added to 200 μL of 50 mM NH_4_HCO_3_ solution at a protein-to-enzyme ratio of 50:1 (w/w) and incubated at 37°C for 16 hours for enzymatic digestion. Following tryptic digestion, peptides were recovered by centrifugation at 14,000 × g for 20 minutes and subsequently dried using a vacuum concentrator. 5% peptides were used to detect the proteome, and 95% peptides were used to prepare phospho-peptides with Fe-NTA enrichment kit (Thermo Scientific, A32992).

#### Enrichment of phosphopeptides and glycopeptides

The peptide sample was fully dissolved in 100 μL of 80% acetonitrile and 0.1% TFA solution following vortex mixing. Subsequently, the precipitation was removed by centrifugation at 16,000 × g for 10 min. The enrichment column was retrieved from the kit (Thermo Scientific, A32992), and the preservation solution was removed by centrifugation at 1,000 × g for 1 min. This was followed by the addition of 100 μL of 80% acetonitrile and 0.1% TFA solution, which was gently mixed and incubated at room temperature for 3 min. After incubation, the solution was removed by centrifugation at 1,000 × g for 1 min, and the peptide solution supernatant was added to the column. The mixture was incubated at room temperature for 30 min, with gentle mixing every 10 min. Following incubation, the peptide solution was removed by centrifugation at 1,000 × g for 1 min. To wash the column, 100 μL of 80% acetonitrile and 0.1% TFA solution was added, and the washing solution was removed by centrifugation, repeating the wash step three times. A clean 1.5 mL Eppendorf tube was then placed beneath the enrichment column, and 100 μL of 50% acetonitrile and 9% ammonia solution was added for elution. The elution was collected by centrifugation at 1,000 × g for 1 min, resulting in the enriched phosphorylated peptide solution. The phosphorylated peptide solution was vacuum-dried, and the peptide powder was stored at -80°C until analysis.

#### RPPA sample processing

The Reverse Phase Protein Array (RPPA) was performed following a standardized workflow to ensure consistency and quality. Protein lysates were initially mixed with a sample dilution buffer (comprising 50% glycerol, 4X SDS buffer, and 6 ml of beta-mercaptoethanol) to achieve a final concentration of 1.5 mg/ml. Normalized samples were then further diluted 2-fold in sample dilution buffer, consisting of lysis buffer, 50% glycerol, and 4X SDS buffer with 6 ml of beta-mercaptoethanol in a 3:4:1 ratio. Five serial dilutions (1, 1/2, 1/4, 1/8, 1/16) were performed using automated liquid handling workstations (Tecan Fluent series). The processed samples were loaded into low-binding 384-well plates (Molecular Devices) and then deposited onto nitrocellulose-coated glass slides (Grace Bio-Labs ONCYTE superNOVA) using a Quanterix 2470 solid pin contact printer.

To maintain quality control (QC), on-slide controls, including treated and untreated cell lines as well as a lysate mixture from various cell lines and tonsil tissue, were applied for staining and quantitative QC checks. Approximately 400 identical slides were prepared for the study.

Each slide was then processed for colorimetric signal quantification using a validated panel of 305 antibodies, targeting 227 total proteins and 78 phosphoproteins or other post-translational modifications (PTMs) (detailed in Table S5). The slides were blocked with Re-Blot (Millipore) at room temperature, followed by I-block (Fisher) and antigen retrieval with hydrogen peroxide (Fisher). Sequential blocking steps with avidin, biotin, and protein block (DAKO) were conducted before a 1-hour primary antibody incubation at room temperature. Secondary antibodies (DAKO) specific for rabbit or mouse were applied, followed by Tyramide Signal Amplification (TSA, Akoya) and DAB colorimetric visualization (DAKO). Staining was fully automated using the DAKO Link 48 Autostainer (Agilent), and the slides were then scanned on a high-throughput slide scanner to capture and analyze colorimetric signals accurately.

#### LC-MS/MS analysis

Dried peptide samples were reconstituted in Solvent A (0.1% formic acid in water) and loaded onto a home-made 100 μm × 2 cm trap column (particle size: 3 μm; pore size: 120 Å; SunChrom, USA) using Solvent A under a maximum pressure of 280 bar. Peptide separation was performed on a home-made 150 μm × 30 cm silica microcolumn (particle size: 1.9 μm; pore size: 120 Å; SunChrom, USA) with a gradient elution of 5–35% mobile phase B (acetonitrile containing 0.1% formic acid) at a flow rate of 300 nL/min over 120 minutes. The eluted peptides were ionized under an applied voltage of 2.2 kV, and mass spectrometry (MS) analysis was conducted in data-dependent acquisition (DDA) mode using an Orbitrap Eclipse mass spectrometer. For global peptide analysis, a precursor scan was acquired in the Orbitrap mass analyzer over an m/z range of 300–1,500 with a resolution of 60,000, followed by MS/MS scans over an m/z range of 200–1,400 at a resolution of 15,000. The most intense ions, selected in top-speed mode, were isolated in the Quadrupole using a 1.6 m/z window and fragmented *via* higher-energy collisional dissociation (HCD) with a normalized collision energy of 32%. The maximum ion injection time was set to 40 ms for full MS scans and 30 ms for MS/MS scans, with a dynamic exclusion time of 30 seconds.

For phosphopeptide analysis, the precursor scan was similarly performed in the Orbitrap mass analyzer over an m/z range of 300–1,500 at a resolution of 60,000, followed by MS/MS scans over an m/z range of 200–1,400, but at an increased resolution of 30,000. The most intense ions, selected in top-speed mode, were isolated in the Quadrupole with a 1.6 m/z window and fragmented using HCD with a normalized collision energy of 27%. The maximum ion injection time was set to 30 ms for full scans and 54 ms for MS/MS scans.

#### Database search

All MS data were processed using the MaxQuant platform (v1.6.17.0). Raw files were searched against the human RefSeq protein database from the National Center for Biotechnology Information (NCBI) (updated on 07-04-2013, containing 32,015 entries). Mass tolerances were set to 10 ppm for precursor ions and 0.05 Da for product ions, allowing up to two missed cleavages. A decoy database search was added, and protein identifications were accepted at a false discovery rate (FDR) of 1%. In the search parameters, carbamidomethylation of cysteine (C) was set as a fixed modification, while acetylation at the protein N-terminus and methionine oxidation (M) were considered variable modifications. For phospho-proteomic database search, the variable modifications included phosphorylation on serine, threonine, and tyrosine. The qualitative analysis of N-glycopeptides from the MS/MS data was conducted using Byonic software version 5.0.3 (Protein Metrics Inc., USA).

#### RPPA analysis

Image data were digitally processed using MicroVigene software (version 5.6.0.8), producing both text (.txt) and image (.tiff) files for each slide. These files were then analyzed using SuperCurve fitting via the R package SuperCurve to produce expression data (rawlog2 files) and quality control (QC) metrics for each slide. Correction factors (CF) were calculated to identify outliers both within and across experiments. For data normalization (to adjust for loading differences), median subtraction was applied: first, each antibody column was median subtracted, followed by median subtraction for each sample row. This resulted in a normalized log2 file, which was squared to create a linear dataset (Normlinear), detailed in Table S5. These processed datasets were then prepared for quantitative comparisons and graphical visualization in downstream analyses.

#### Data processing

MS-based proteomics and phosphoproteomics data were analyzed using MaxQuant's label-free quantitation (LFQ) approach to assess protein and phosphorylation-site abundances. Prior to further processing, proteins, and phosphorylation sites present in less than 5% of samples were filtered to account for the broad lineage diversity of cell lines, thus applying a more lenient threshold than usual. Data were manually examined using the R package "DEP" (v1.16.0). Data visualization and preprocessing were performed with "Seurat" (v5.0.1), using the "NormalizeData" function with the "LogNormalize" method. The R package "COSG" was utilized to identify marker proteins, setting a COSG score threshold of 0.5. The protein change calculations were based on comparing the mean of selected cell lines to the mean of all other cell lines. For calculating the fold change (FC) of protein expression and phosphorylation sites, the "FindAllMarkers" function in "Seurat" was applied.

For mass spectrometry-based glycoproteomics, each glycopeptide is annotated with its glycan composition, which includes sialic acid (NeuAc), fucose (Fuc), N-acetylhexosamine (HexNAc), and hexose (Hex). A site-specific glycan is defined as a particular type of glycan located at a specific glycosylation site on a protein. This definition encompasses peptides of varying lengths, including those with missed cleavages and differing charge states of the peptide ions. The abundance of each site-specific glycan is estimated using spectral counting, which involves summing the number of spectra corresponding to all peptide ions that contain this type of glycan at the designated sites. The R code used for this analysis is provided in Data S1.

For RPPA data, antibody probes were mapped to Uniprot Accession numbers of target proteins. In cases of multiple mappings, a representative protein was selected to facilitate comparisons between RPPA and MS data.

To enable comparisons across data modalities, the data were processed systematically for consistency across platforms. For MS proteomics and phosphoproteomics, raw abundance values were log2(x+1)-transformed, followed by averaging replicate values to quantify each cell line. In the case of MS glycoproteomics, site-specific peptide counts for each modification, site, or protein were averaged across replicates for cell line quantification. For RPPA proteomics and phosphoproteomics, mean normalized expression values were calculated from replicates to quantify each cell line. Correlation analyses were performed using these standardized quantified values to examine relationships across cell lines.

#### Functional annotation, enrichment analysis, kinase activity estimation, and gene correlation analysis

GO annotations were performed using the "org.Hs.eg.db" R package (v3.18.0), while KEGG pathway data were accessed through "KEGGREST" (October 11, 2024). GO enrichment analysis was carried out using "clusterProfiler" (v4.10.0). To annotate the function of detected proteins of MS and RPPA data, all 3 GO categories were used. Enriched results were further processed by "simplify" function in "clusterProfiler" package to eliminate the redundancy of GO terms.

KSEA (Kinase-Substrate Enrichment Analysis) was conducted using "KSEAapp" (v0.99.0) to estimate kinase activity changes by averaging multiple substrate measurements rather than single-substrate dependence. Fold change (FC) values for phosphorylation sites in each cell line were computed as previously described. Kinase-substrate relationships were sourced from PhosphoSitePlus® (January 15, 2024). A Z-score was calculated by comparing the mean log2(FC) of each kinase’s substrate phosphosites against the mean log2(FC) of all phosphosites in that cell line. Kinases with significant Z-scores (FDR < 0.05) and more than five substrates were considered to exhibit altered activity.

To visualize the distribution of identified kinases across families, a kinome tree was generated using KinMap,^61^ which maps detected kinases onto a phylogenetic tree of the human kinome, enabling interpretation of their classification and functional groupings.

Gene-level correlations were assessed using the multiOmicsViz R package. CNA data were sourced from the OmicsCNGene.csv file (CCLE 2023 Q2 release)^14^ and quantified as log_2_(CN ratio + 1). Proteomic intensities were normalized to total protein abundance per sample. Phosphosite intensities were adjusted by corresponding protein levels to account for expression variability. Spearman correlations between CNA and proteomic or phosphoproteomic features were computed to visualize cis- and trans-regulatory effects genome-wide.

### Quantification and statistical analysis

Quantification strategies for proteome, phosphoproteome, glycoproteome, and RPPA data were detailed in the sections above. Briefly, MaxQuant LFQ intensities and spectral counts were used for MS data, and SuperCurve-normalized intensities for RPPA.

For statistical analysis, features detected in fewer than 5% of cell lines were excluded. Missing values were imputed with the global minimum observed intensity, reflecting undetected low-abundance peptides. MS data were log_2_(x+1)-transformed and normalized by sample-wise median adjustment. RPPA data were log_2_-transformed, median-centered, and squared.

All statistical analyses were performed in R (version 4.3.2). Unless otherwise specified, Spearman’s rho was used for correlation analyses due to variations in scale and distribution across modalities. For differential analysis of MS-based proteomics data, the Wilcoxon rank-sum test was applied. P-values were adjusted using the Bonferroni correction based on the total number of proteins in the dataset. Proteins with an average |log_2_FC| > 2 and an adjusted P-value < 0.05 were considered differentially expressed. For KSEA and GO enrichment analyses, the Benjamini–Hochberg (BH) method was used for P-value adjustment.

## Supplemental information

Table S1. Panel of 54 cancer cell lines with lineage, disease, subtype, and protein identifiers

Table S2. MS total proteome profiles and tissue-specific protein markers

Table S3. MS phosphoproteome matrix with localization, sequence context, and annotations

Table S4. MS glycoproteome matrix with site-specific glycans and annotations

Table S5. RPPA antibody panel with protein and phospho-site targets

Data S1. R script for site-specific glycan quantification
